# BAY 11-7085 induces glucocorticoid receptor activation and autophagy that collaborate with apoptosis to induce human synovial fibroblast cell death

**DOI:** 10.18632/oncotarget.8042

**Published:** 2016-03-14

**Authors:** Biserka Relic, Edith Charlier, Celine Deroyer, Olivier Malaise, Sophie Neuville, Aline Desoroux, Philippe Gillet, Dominique de Seny, Michel G. Malaise

**Affiliations:** ^1^ Department of Rheumatology, GIGA Research, University Hospital Sart-Tilman, Liege, Belgium; ^2^ Department of Orthopedic Surgery, University Hospital Sart-Tilman, Liege, Belgium

**Keywords:** BAY 11-7085, autophagy, cell death, glucocorticoid receptor, PPAR-γ

## Abstract

Inhibition of proapoptotic pathways in synovial fibroblasts is one of the major causes of synovial proliferation and hyperplasia in rheumatic diseases. We have shown previously that NF-κB inhibitor BAY 11-7085, through inactivation of PPAR-γ, induces apoptosis in human synovial fibroblasts. In this work we showed that BAY 11-7085 induced autophagy that preceded BAY 11-7085-induced apoptosis. Of interest, BAY 11-7085 induced Serine 211 phosphorylation and degradation of glucocorticoid receptor (GR). Glucocorticoid prednisolone induced both activation and degradation of GR, as well as autophagy in synovial fibroblasts. BAY 11-7085-induced cell death was significantly decreased with glucocorticoid inhibitor mifepristone and with inhibitors of autophagy. Both BAY 11-7085-induced autophagy and GR activation were down regulated with PPAR-γ agonist, 15d-PGJ2 and MEK/ERK inhibitor UO126. Inhibition of autophagy markedly decreased endogenous and BAY 11-7085-induced ERK phosphorylation, suggesting a positive feed back loop between ERK activation and autophagy in synovial fibroblasts. Co-transfection of MEK1 with PPAR-γ1 in HEK293 cells caused known inhibitory phosphorylation of PPAR-γ1 (Serine 112) and enhanced GR degradation, in the absence or presence of prednisolone. Furthermore, GR was both phosphorylated on Serine 211 and down regulated in synovial fibroblasts during serum starvation induced autophagy. These results showed that GR activation and PPAR-γ inactivation mediated BAY 11-7085-induced autophagy.

## INTRODUCTION

Autophagy, the cell process that degrades unused cytoplasmic components and damaged organelles, relieves cells from stress and represent an important catabolic process that maintains cellular energy level during starvation [1]. In rheumatoid arthritis (RA), autophagy is deregulated and can favor synovial fibroblast cell survival or synovial fibroblast cell death [2]. Furthermore, autophagy is found to be more prominent in synovial fibroblasts from RA patients than in synovial fibroblasts from osteoarthritis (OA) patients and represents one of the markers for OA to RA transition *in vitro* [3]. However, autophagy has a dual role in rheumatic diseases [2] because of its protective effect on cartilage [4, 5]. Enhanced autophagy, through inhibition of mTOR has been proposed as an effective treatment in OA [4, 6, 7]. Furthermore, glucocorticoids, one of the most powerful anti-inflammatory drugs used in rheumatic diseases, have been shown as autophagic inducers in chondrocytes, although their prolonged application led to autophagy inhibition [8].

Autophagy mediates cell fate through the cross-talk with apoptosis [9]. In some cases autophagy can protect cells from apoptosis [8], while autophagy can also act as agonist of apoptosis [10]. Our previous work showed that BAY 11-7085, a potent NF-κB inhibitor, induces apoptosis in human synovial fibroblasts, through inactivation of PPAR-γ [11, 12]. PPAR-γ, a transcription factor involved in adipocyte differentiation, fatty acid storage and glucose metabolism, is involved in metabolic diseases such as obesity and diabetes [13]. However, PPAR-γ is also involved in cell survival and apoptosis [10–12], as well as in autophagy [10]. Thus, in this work we have studied possible involvement of autophagy in BAY 11-7085-induced human synovial fibroblasts apoptosis.

## RESULTS

### BAY 11-7085 induces the increase of LC3B-II, GR phosphorylation on Serine 211, as well as GR down regulation in human synovial fibroblasts

**Figure 1:**
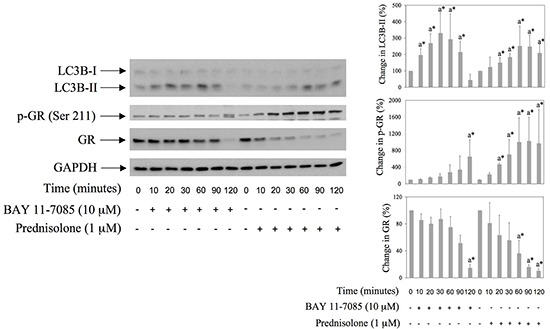
BAY 11-7085 and prednisolone induce autophagy, GR phosphorylation and GR down regulation in human synovial fibroblasts Cells were cultured with BAY 11-7085 or prednisolone for the time indicated. Western blots show expression of p-GR (Serine 211), GR, LC3B and GAPDH in synovial fibroblast cell extracts. Graphs represent average of protein expression from three different experiments done with synovial fibroblasts from three different OA patients, expressed as a percentage of control. a* statistically different from control cells.

Glucocorticoids are known inducers of autophagy in several cell types [14, 15]. To study possible involvement of autophagy in BAY 11-7085-induced apoptosis, we have monitored the expression of LC3B, a marker of autophagy, in BAY 11-7085- or prednisolone-treated synovial fibroblasts, from 10-120 minutes (Figure 1). Western blot have shown that BAY 11-7085 induced an increase of autophagosomal marker LC3B-II, a lipidated form of LC3B, from 10-60 minutes, that ceased and that was down regulated from 90-120 min of cell treatment (Figure 1). Prednisolone induced increase of LC3B-II from 20-120 minutes, suggesting autophagy. Prednisolone also markedly induced GR phosphorylation on Serine 211 as well as GR down regulation (Figure 1). Of interest, similarly to prednisolone, BAY 11-7085 induced GR phosphorylation of Serine 211 (Figure 1). Furthermore, as prednisolone, BAY 11-7085 markedly down regulated GR expression (Figure 1). These results suggested GR activation and GR autoregulation that was shown earlier [16]. Furthermore, these results suggested that BAY 11-7085-induced autophagy and GR activation might be interconnected.

### BAY 11-7085-induced autophagy collaborates to human synovial fibroblast apoptosis

**Figure 2:**
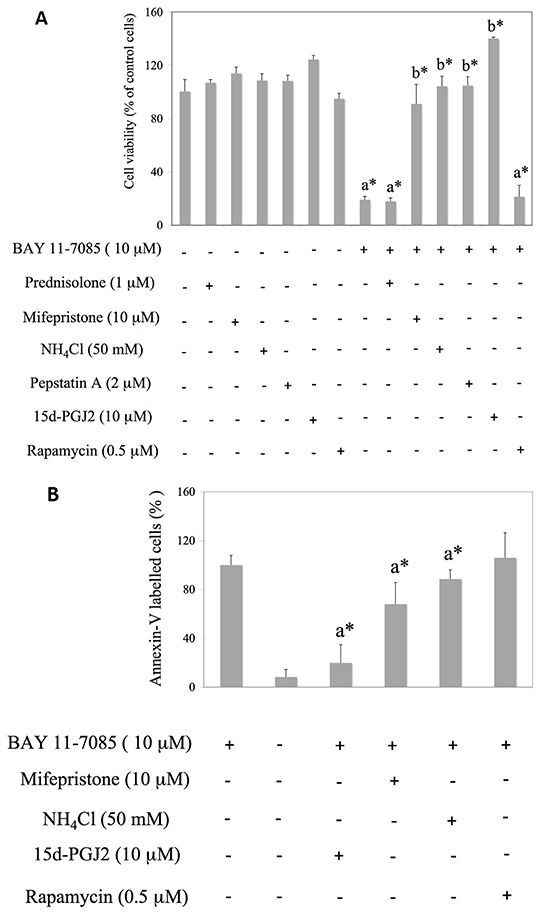

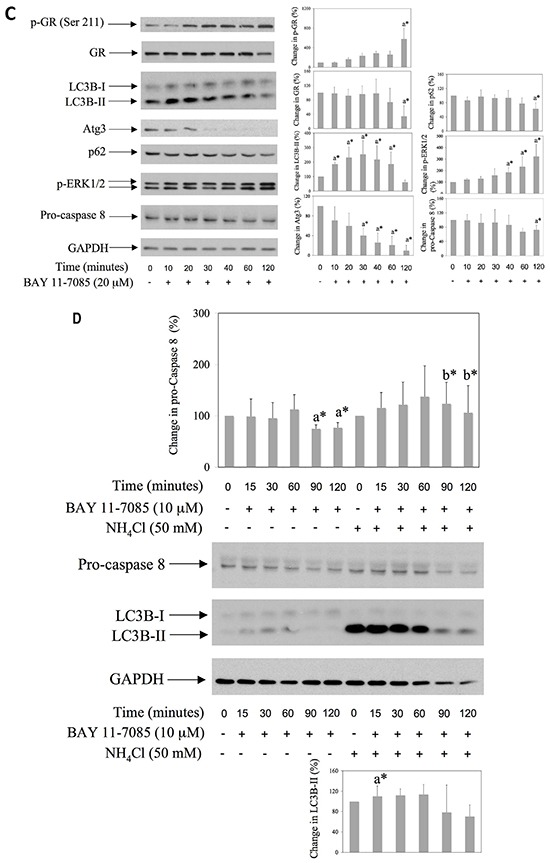
Mifepristone and inhibitors of autophagy protect synovial fibroblasts from BAY 11-7085-induced apoptosis Synovial fibroblasts were cultured with GR agonist prednisolone, GR inhibitor mifepristone, inhibitors of autophagy NH_4_Cl and Pepstatin A or mTOR inhibitor rapamycin for 24 hours and BAY11-7085 was then added for additional 2 hours. 15d-PGJ2 was used as a positive control [11]. **A.** Cell survival, estimated by an MTS test, was expressed as a percentage of surviving cells compared with control cells. a* statistically different from control cells. b* statistically different from BAY 11-7085 treated cells. **B.** Annexin-V binding was determinated by FACS and results are presented as % of Annexin-V binding to BAY 11-7085 treated cells. Graphs represent average of four different experiments done with synovial fibroblasts from three different OA patients. a* statistically different from BAY 11-7085 treated cells. **C.** Synovial fibroblasts were cultured in the presence of BAY 11-7985 for the time indicated. Western blots show expression of p-GR (Ser 211), GR, LC3B, p-ERK1/2, Atg3, p62, pro-Caspase 8 and GAPDH in synovial fibroblast cell extracts. Graphs represent average of protein expression from three different experiments done with synovial fibroblasts from three different OA patients, expressed as a percentage of control. a* statistically different from control cells. **D.** Synovial fibroblasts were pretreated or not with NH_4_Cl for 2 hours and then BAY 11-7085 was added for the time indicated. Western blots show expression of LC3B, pro-Caspase 8 and GAPDH in synovial fibroblast cell extracts. Graphs represent average of protein expression from three different experiments done with synovial fibroblasts from three different OA patients, expressed as a percentage of control. a* statistically different from control cells: b* statistically different from a*.

To test the possible involvement of autophagy in BAY 11-7085-induced apoptosis, we have pretreated synovial fibroblasts with GR agonist prednisolone, GR inhibitor mifepristone, mTOR inhibitor rapamycin or inhibitors of autophagy, *i.e.* NH_4_Cl or Pepstatin A for 24 hours. BAY 11-7085 was then added for additional 2 hours (Figure 2A). PPAR-γ agonist 15d-PGJ2 was used as a positive control [11]. MTS test showed that inhibitors of autophagy, as well as GR inhibitor mifepristone, significantly decreased BAY 11-7085-induced cell death (Figure 2A). BAY 11-7085 induced intensive Annexin-V binding in synovial fibroblasts that was significantly diminished by 15d-PGJ2, mifepristone and NH_4_Cl, but not with rapamycin pretreatment (Figure 2B). These results indicated that both autophagy and GR activation potentiated BAY 11-7085-induced cell death. Furthermore, Western blot showed that increase of LC3B-II in the BAY 11-7085 treated synovial fibroblasts preceded both the cleavage of pro-Caspase 8 and ERK1/2 phosphorylation [11] (Figure 2C). Furthermore, preincubation of cells with autophagy inhibitor NH_4_Cl significantly prevented pro-Caspase 8 downregulation and showed that autophagy LC3B flux preceded pro-Caspase 8 down regulation (Figure 2D). These results also suggested that BAY 11-7085-induced autophagy has pro-apoptotic effect in synovial fibroblasts. However, timing experiments also showed that BAY 11-7085-induced autophagy might be exhaustive since down regulation of LC3B and Atg3, an E2 enzyme involved in LC3B lipidation, was observed after 30 min of BAY 11-7085 treatment (Figure 2C).

### 15d-PGJ2 inhibits BAY 11-7085 induced GR phosphorylation on Ser 211, down regulation of GR expression and autophagy in synovial fibroblasts

**Figure 3:**
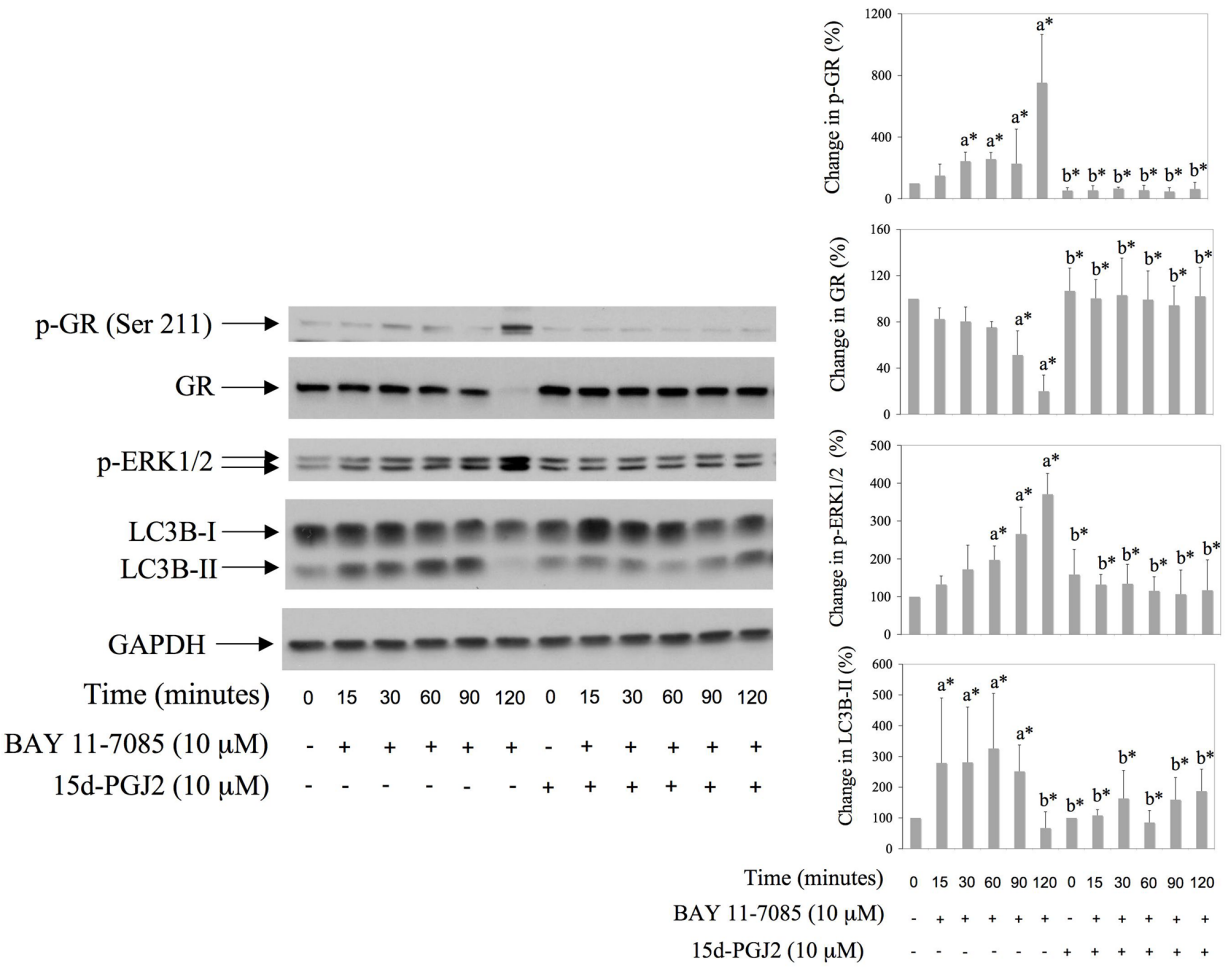
BAY 11-7085 induced autophagy, GR phosphorylation on Serine 211 and GR degradation are inhibited with PPARγ agonist 15d-PGJ2 Synovial fibroblasts were pretreated with 15d-PGJ2 for 24 hours and then BAY 11-7985 was added for the time indicated. Western blots show expression of p-GR (Ser 211), GR, p-ERK1/2, LC3B and GAPDH in synovial fibroblast cell extracts. Graphs represent average of protein expression from three different experiments done with synovial fibroblasts from three different OA patients, expressed as a percentage of control. a* statistically different from control cells; b* statistically different from a*.

PPAR-γ agonist 15d-PGJ2 is a potent inhibitor of BAY 11-7085-induced apoptosis in synovial fibroblast [11]. We tested the effect of 15d-PGJ2 on BAY 11-7085-induced autophagy, GR phosphorylation on Serine 211 and GR down regulation. Synovial fibroblasts were cultured in the presence or absence of 15d-PGJ2 for 24 hours and then BAY 11-7085 was added for additional 15-120 minutes (Figure 3). Western blots showed that 15d-PGJ2 inhibited BAY 11-7085-induced GR phosphorylation on Serine 211 and GR down regulation, as well as BAY 11-7085 induced LC3B-II (Figure 3). These results strongly suggested that PPAR-γ agonist 15d-PGJ2 protected synovial fibroblast from BAY 11-7085-induced cell death through autophagy inhibition.

### MEK/ERK inhibitor UO126 down regulates BAY11-7085-induced autophagy and delays BAY 11-7085-induced GR down regulation

**Figure 4:**
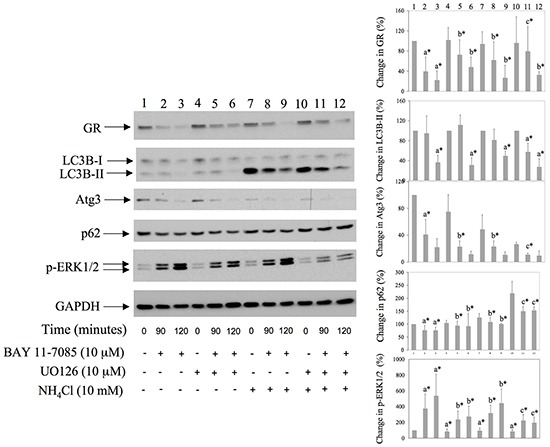
MEK/ERK inhibitor down regulates BAY 11-7085-induced autophagy and protects GR from BAY11-7085-induced degradation Synovial fibroblasts were pretreated with MEK/ERK inhibitor UO126 for 24 hours. BAY 11-7085 (20 mM) was then added, alone or simultaneously with inhibitor of autophagy NH_4_Cl, for the time indicated. Western blots show expression of GR, LC3B, Atg3, p62, p-ERK1/2, and GAPDH in synovial fibroblast cell extracts. Graphs represent average of protein expression from three different experiments done with synovial fibroblasts from three different OA patients, expressed as a percentage of control. a* statistically different from control cells; b* statistically different from a*; c* statistically different from b*. Numbers on the top of graphs correspond to the Western lane numbers.

We have shown previously that BAY 11-7085-induced ERK1/2 phosphorylation participates in BAY 11-7085-induced apoptosis [11, 12] and that MEK/ERK inhibitor UO126 partially protects synovial fibroblasts from BAY 11-7085-induced apoptosis [12]. Since ERK1/2 phosphorylation followed BAY 11-7085-induced increase of LC3B-II (Figure 2C and Figure 3), we have tested the effect of UO126 on BAY 11-7085-induced autophagy. Synovial fibroblasts were pretreated with UO126 for 24 hours and then with BAY 11-7085, in the presence or absence of inhibitor of autophagy NH_4_Cl, for 90-120 minutes (Figure 4). Results showed that UO126, in parallel with inhibition of BAY 11-7085-induced ERK1/2 phosphorylation, inhibited BAY 11-7085-induced p62 down regulation, and, of interest, delayed GR degradation (Figure 4). In addition, UO126 further declined BAY 11-7085-induced Atg3 down regulation. Furthermore, these effects of UO126 were mimicked with inhibitor of autophagy NH_4_Cl that, of interest, alone or in synergy with UO126 decreased endogenous and BAY 11-7085-induced ERK1/2 phosphorylation (Figure 4). These results showed that ERK activation is involved in BAY 11-7085-induced autophagy and suggested a feed back loop between ERK activation and autophagy. Furthermore, these results suggested that autophagy might be, at least partially, involved in BAY 11-7085-induced GR down regulation.

### Co-transfection of MEK1 and PPARγ induce GR degradation in HEK293 cells

**Figure 5:**
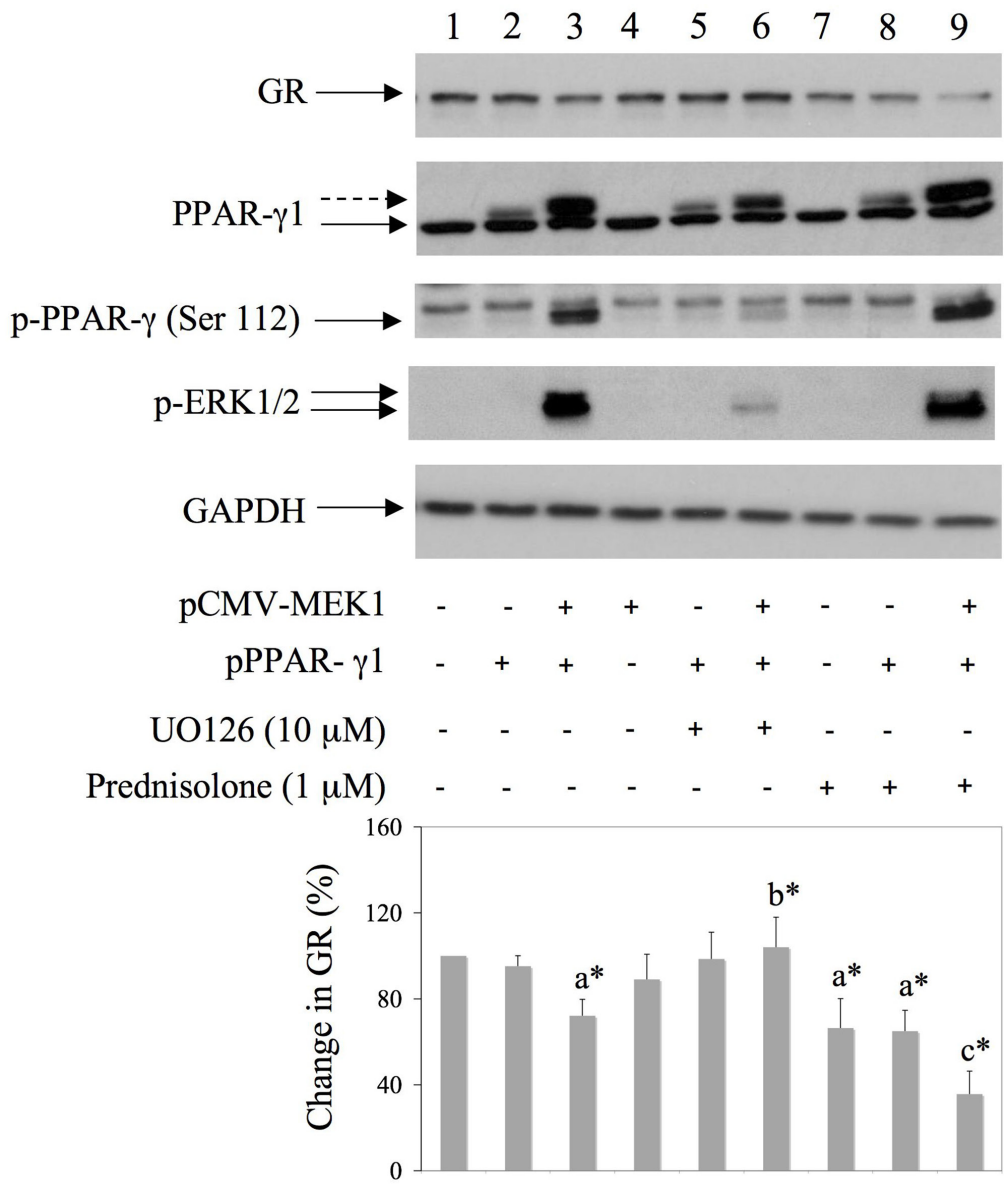
Co-transfection of MEK1 and PPAR-γ down regulates GR expression in HEK293 cells HEK293 were transfected with pMEK1 [17] and pPPAR-γ1 [12] and 16 hours later UO126 or prednisolone were added for additional 8 hours. Western blots show GR, PPARγ, PPARγ phosphorylation on Serine 112 and GAPDH expression in HEK293 cell extracts. Graph represents average of protein expression from three different experiments, expressed as a percentage of control. a* statistically different from control cells; b* statistically different from a*; c* statistically different from a*.

Since BAY11-7085-induced GR down regulation was inhibited by PPAR-γ agonist 15d-PGJ2 (Figure 3), we have tested involvement of PPAR-γ in GR down regulation. To test that hypothesis we have expressed pCMV-MEK1 [17] and/or pPPAR-γ1 [12] in HEK293 cells and monitored GR expression by Western blot (Figure 5). Phosphorylation of PPAR-γ by ERK has been reported previously to inhibit PPAR-γ function by phosphorylation on Serine 112 [18] [19]. Results showed that prednisolone alone significantly down regulated GR in HEK293 cells (Figure 5, lane 7). Co-expression of pCMV-MEK1 with pPPAR-γ1 induced PPAR-γ phosphorylation on Serine 112 as well as GR degradation, in the presence or absence of prednisolone (Figure 5, lanes 3 and 9). Furthermore, the effect of PPAR-γ phosphorylation on GR degradation was not significant when cells were treated with MEK/ERK inhibitor UO126 (Figure 5, lane 6). These results suggested that inactivation of PPAR-γ is involved in GR down regulation.

### Starvation induces both autophagy and GR activation in human synovial fibroblasts

**Figure 6:**
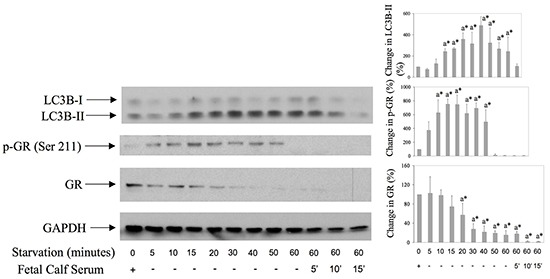
GR is both phosphorylated on Serine 211 and down regulated during starvation-induced autophagy in synovial fibroblsts Cells were first starved by serum deprivation from the culture medium for 5-60 minutes, and then serum (10%) was replaced or not for additional 5-15 minutes. Western blots show expression of p-GR (Ser 211), GR, LC3B and GAPDH in synovial fibroblast cell extracts. Graphs represent average of protein expression from three different experiments done with synovial fibroblasts from three different OA patients, expressed as a percentage of control. a* statistically different from control cells.

To test further the possible role of GR activation and down regulation in autophagy, we induced autophagy in human synovial fibroblasts by 5-60 minutes starvation (Figure 6). Results showed that, in parallel with autophagosomal marker LC3B-II, starvation also induced GR phosphorylation on Serine 211 as well as GR down regulation (Figure 6). These results further suggested the involvement of GR in synovial fibroblast autophagy.

## DISCUSSION

BAY 11-7085 is an inhibitor of IκBα phosphorylation that leads to NF-κB inactivation and inflammation down regulation in mouse model of asthma [20, 21], in articular chondrocytes [22, 23], as well as in synovial fibroblasts [24]. We have shown previously that BAY 11-7085 induces sustained ERK phosphorylation and apoptosis in human articular chondrocytes and synovial fibroblasts [11]. Furthermore, we have shown that inactivation of PPAR-γ was necessary for BAY 11-7085-induced synovial fibroblast apoptosis [12]. In this work, we showed that BAY 11-7085 induced autophagy that is involved in, and that favored BAY 11-7085-induced apoptosis. BAY 11-7085-induced autophagy, through the inhibition of NF-κB, has been reported in macrophages [25]. In our experimental model, BAY 11-7085-induced autophagy included ERK and GR activation, as well as PPAR-γ inactivation. Recently, it was shown that ERK activation could regulate expression levels of LC3B and SQSTM1/p62 [26]. ERK activity is also needed for autophagy in the Nara-H malignant fibrous histiocytoma cell line and in these cells rapamycin induces autophagy, through p-ERK, as well as apoptosis [27]. In these cells, upon UO126 treatment, apoptosis is enhanced, while autophagy is diminished. However, our previous results showed that UO126 can protect synovial fibroblasts from apoptosis [12], and in this work, we have shown that UO126 inhibited BAY 11-7085-induced autophagy. Thus, it is likely that UO126 protects synovial fibroblasts from BAY 11-7985-induced cells death through autophagy inhibition. Of interest, we have also shown here that inhibition of autophagy markedly decreased endogenous and BAY 11-7085-induced ERK phosphorylation and that LC3B-II upregulation preceded ERK phosphorylation, suggesting that autophagy induced ERK phosphorylation. These results suggested the positive feed back loop between autophagy and ERK phosphorylation in synovial fibroblasts. It has been reported recently that ERK activation is involved in autophagy [28, 29], as well as that autophagy proteins are implicated in ERK phosphorylation [30, 31]. Furthermore, it is known that p62 k/o mice have an increase in p-ERK [32]. Because of pro-inflammatory role MEK/ERK in rheumatic diseases [33], its inhibition may down regulate both inflammation and autophagy. However, autophagy is described as cell-protected phenomena for articular chondrocytes [4, 7, 8, 34]. In addition, UO126 can prevent PTH positive effects on intervertebral disc calcification [35]. Although UO126 has been reported to decrease a pain in inflamed joint of rats [36], the use of MEK/ERK inhibition in the human joint must be elucidated with precaution. In contrast, stimulation of autophagy, shown to be beneficial for cartilage [4, 7, 8], might also prevent synovial hyperplasia by favoring synovial cell death, as suggested by our results presented here.

PPAR-γ is a transcription factor involved in adipocyte differentiation, fatty acid storage and glucose metabolism and its deregulation is involved in metabolic diseases such as obesity and diabetes [13]. Known anti-diabetic drugs such as rosiglitazone are ligands of PPAR-γ [37]. However, PPAR-γ is also involved in cell survival and apoptosis, and we have shown previously that PPAR-γ is anti-inflammatory and anti-apoptotic protein in synovial fibroblasts [11, 12]. In mouse prostatic cells, disruption of PPAR-γ activates autophagy [38] and PPAR-γ inhibits autophagy in human monocyte-derived macrophages [39]. Results of this work also showed that PPAR-γ is inhibitor of autophagy in synovial fibroblasts because PPAR-γ agonist, 15d-PGJ2, stabilized LC3B-I and prevented BAY 11-7085-induced autophagy. Of interest, 15d-PGJ2 also prevented BAY 11-7085-induced GR phosphorylation on Serine 211 as well as BAY 11-7085-induced GR down regulation in synovial fibroblasts. Glucocorticoids are known inducers of autophagy in several experimental systems. Dexamethasone and cortisol induce autophagy in T lymphocytes [14] and HeLa cells [15], respectively. Furthermore, dexamethasone-induced autophagy leads to muscle atrophy [40]. However, glucocorticoids have dual effect on chondrocytes, short time exposure induces autophagy while long time exposure can lead to autophagy inhibition [8]. In addition dexamethasone induces autophagy in osteocytes [41]. Furthermore, autophagy was proposed as a main pathway in TNF-α-induced bone loss [42]. In this work we showed that prednisolone induced autophagy in synovial fibroblasts, as well as GR phosphorylation on Serine 211, that has been shown to activate GR [43] and GR down regulation, a known GR auto regulation [16]. However, we have observed that longer exposure of synovial fibroblasts to prednisolone leads to stabilization of LC3B-I and LC3B-II, suggesting autophagy inhibition (data not shown). These results suggested that glucocorticoid effects on the joint derived cells might be time dependent [8]. GR degradation is known to be auto regulated [16] and proteasome dependent [44]. Our results presented here suggested that autophagy might be also involved in GR down regulation because GR degradation was partially prevented with 15d-PGJ2, UO126 and NH_4_Cl that simultaneously inhibited autophagy. Since both GR phosphorylation on Serine 211 and GR down regulation were induced, in parallel with autophagy, with BAY 11-7085, prednisolone and by cell starvation, our results suggested a prominent role of GR in synovial fibroblast autophagy. Our results also suggested that ERK activation and inactivation of PPAR-γ are involved in GR down regulation because the co-transfection of PPAR-γ and MEK1 led to PPAR-γ phosphorylation on Serine 112 and enhanced GR degradation. Phosphorylation of PPAR-γ by ERK on Serine 112 was reported as inhibitory [45]. Thus our results suggested that MEK1, through the ERK1/2 activation, inhibited PPAR-γ by Serine 112 phosphorylation and that inactivation of PPAR-γ may activate autophagy that might be involved in GR turnover. However, inactivation of PPAR-γ acted in synergy with prednisolone-induced GR down regulation in HEK293 cells, suggesting that GR down regulation may dependent on autophagy induced by two different mechanisms. These effects, together with effect of synovial fibroblasts starvation, that also induced autophagy that coincided with GR down regulation, further suggest an involvement of autophagy in GR down regulation.

We showed here that BAY 11-7085-induced autophagy (increase of LC3B-II expression and p62 degradation [46]) was followed by decrease of Atg3 expression, suggesting that BAY 11-7085 induced exhaustive autophagy in synovial fibroblasts. Decrease of Atg3 expression might result from Caspase 8 action [47]. Furthermore, cleavage of Caspase 8 followed LC3BII up regulation and Atg3 down regulation. Atg3 is also known to regulate cell fate [48] and in human airway mesenchymal cells inactivation of Atg3 led to increased simvastatin-induced apoptosis [49]. BAY 11-7085-induced autophagy coincided with BAY 11-7085-induced GR phosphorylation as well as GR down regulation, a phenomena that also followed both prednisolone- and starvation-induced autophagy in synovial fibroblasts. However, it is unlikely that BAY 11-7085 has a direct effect on GR. Our hypothesis is that inactivation of PPAR-γ by BAY 11-7085 leads to GR activation and autophagy that might be involved in GR turnover.

In our experimental system 15d-PGJ2 efficiently protected synovial fibroblasts from BAY 11-7085- induced cell death in both MTS (a proliferation assay based on mitochondria activity) and Annexin V labelling (an apoptosis assay that measure phosphatidyl-serine externalization) assays. However, cell protection with both mifepristone and NH_4_Cl, although significant in both assays, was more efficient in MTS assay. These results suggest that inhibition of autophagy protected synovial fibroblasts more from BAY 11-7085-induced mitochondrial dysfunction and/or mitochondrial injury than from BAY 11-7085-induced apoptosis. We have detected autophagy markers earlier in the timing experiments then apoptosis markers, and inhibitor of autophagy partially prevented apoptosis. In mammary carcinoma cells, it has been shown that doxorubicin causes mitochondrial injury that precedes phosphatidyl-serine externalization [50]. Taken together, our results suggested that BAY 11-7085-induced autophagy and apoptosis in human synovial fibroblasts partially overlap, *i.e.* that apoptosis is partially the consequence of autophagy and that both autophagy and apoptosis are participating in BAY 11-7085-induced cell death. The difference between 15d-PGJ2 effect and NH_4_Cl and mifepristone effects may lay in the ability of 15d-PGJ2 that, in addition to autophagy inhibition, also inhibits apoptosis by another PPAR-γ signalling pathway. Thus, while NH_4_Cl and mifepristone are suggested to act through autophagy inhibition, PPAR-γ may be also one of the common regulators of both autophagy and apoptosis, as it was very recently proposed for Gadd45b (Growth arrest and DNA damage response 45b gene) [51].

In summary, this work showed that BAY 11-7085 induced human synovial fibroblast autophagy that act as an agonist to promote BAY 11-7085-induced apoptosis. Furthermore, our results suggested that BAY 11-7085-induced autophagy in synovial fibroblasts is mediated through GR activation. We have shown previously that, in synovial fibroblasts, glucocorticoids also have inflammatory properties such as stimulation of leptin and Ob-R expression [52, 53]. Thus, modulation of autophagy and inflammation through regulation of GR expression is a challenging approach to be further studied in rheumatic diseases.

## MATERIALS AND METHODS

### Cell isolation and culturing

Primary human synovial fibroblasts from osteoarthritis patients were isolated and cultured as explained previously [11]. For experiments, 5 × 10^4^ cells were seeded in 24 well plates (BD Biosciences, San Jose, CA), within 0.5 ml of culturing medium consisting of DMEM medium (Cambrex Bio Science, Walkersville, MD, USA) supplemented with 10% FCS (Lonza, Basel, Switzerland), L-glutamine (2mM), streptomycin (100 mg/ml) and penicillin (100 U/ml) (BioWhittaker, Walkersville, MD, USA), in triplicates. All experiments were repeated at least three times with synovial fibroblasts from at least three different donors.

### Survival assay

Synovial fibroblasts were stimulated with 15d-PGJ2 (BioMol, Plymouth Meeting, PA), prednisolone, mifepristone, pepstatin A (Sigma-Aldrich, St. Louis, MO), rapamycin (Calbiochem, San Diego, CA), or NH_4_Cl (Acros Organics) for 24 h and then BAY 11-7085 (Alexis Corp., San Diego, CA) was added for additional 2h. Survival assay, MTS (Promega, Madison, WI), was performed for additional 1h, as explained earlier [11].

### FACS analysis

Synovial fibroblasts were seeded in 6 well plate and pretreated with 15d-PGJ2, mifepristone, NH_4_Cl or rapamycin, in triplicates, for 24 hours. The following day BAY11-7085 was added for additional 2 hours. Cells were collected by combination of gentle trypsinisation and scrapping, centrifuged, resuspended in Annexin-V/PI from Annexin-V-Fluos staining kit (Roche Diagnostics GmbH, Mannheim, Germany) and analyzed by FACS (FACSCanto II) and FACSDiva software (BD Biosciences, Franklin Lakes, NJ).

### Timing experiments

Synovial fibroblasts were pretreated with 15d-PGJ2 or UO126 (Cell Signaling, Beverly, MA) for 24 h and then prednisolone, NH_4_Cl, and/or BAY 11-7085 were added for additional 10-120 minutes.

### Cell starvation

Starvation was done by serum deprivation for 5-60 minutes.

### Western blotting

Cells were collected on ice in lysis buffer [12], and total proteins separated by SDS-PAGE as explained previously [11]. Membranes were incubated with LC3B antibody (L7543) (Sigma-Aldrich), atg3 (#3415), p62 (PW9860) (Enzo Life Sciences, Inc., Ann Arbor, MI), glucocorticoid receptor GR (41) (sc-136209) (Santa Cruz Biotechnology, Santa Cruz, CA), phospho-glucocorticoid receptor pSer211 (PA5-17668) (Thermo Scientific, Pittsburgh PA), PPAR-γ (H-100; sc-7196) (Santa Cruz Biotechnology), phospho-PPAR-γ pSer112 (PA5-35664) (Thermo Scientific), phospho-ERK1/2 on Tyr-204 (E4; sc-7383) (Santa Cruz Biotechnology), Caspase 8 (804-429) (ENZO) and GAPDH (G9545) (Sigma-Aldrich) antibodies for 1 hour. Western blots were shown with 1:1000 diluted anti-mouse or anti-rabbit (Cell Signaling) antibodies and ECL Western blotting substrate (Thermo Scientific). Western blots were scanned with Image Studio Lite Software (Li-Cor Biosciences, Linkolin, Nebraska, NE) and normalized by GAPDH values.

### Plasmids and DNA transfection

pPPAR-γ1 [12], pCMV-MEK1 [17] (a kind gift from Dr Kun-Liang Guan, University of Michigan) and pEGFP (Clontech Laboratories Inc., Mountain View, CA) were over expressed in HEK293 cells. For transfection, 28 × 10^4^ cells were seeded in 6 well plates (BD Biosciences), within 2ml of culturing medium. Cells contained in each well were transfected with 3 mg of DNA by the use of linear polyethylenimine (MW 25,000) (Polysciences, Inc., Warrington, PA).

### Statistical analysis

*p* values were obtained using the Mann-Whitney test and Student's *t*-test. A value of p < 0.05 was considered as statistically significant.
